# Genetic Diversity and Structure of an Endangered Medicinal Plant Species in the Northern Balruk Mountains: Implications for Conservation

**DOI:** 10.3390/genes17091010

**Published:** 2026-08-26

**Authors:** Zhihao Su, Tiangang Hu, Li Zhuo, Kaiqing Xie, Qichuan Jiang

**Affiliations:** 1 Xinjiang Key Laboratory of Special Species Conservation and Regulatory Biology, College of Life Science, Xinjiang Normal University, Urumqi 830017, China; 2 Library, Xinjiang Normal University, Urumqi 830054, China; 3 Key Laboratory of Bioinformatics and Computational Biology of the Department of Education of Yunnan Province, Dali University, Dali 671003, China; 4 Yunan Key Laboratory of Forest Plant Cultivation and Utilization, Yunnan Academy of Forestry and Grassland, Kunming 650204, China

**Keywords:** *Fritillaria karelinii*, SNP, genetic diversity, genetic structure, conservation

## Abstract

**Background**: *Fritillaria karelinii* is an endangered medicinal herb mainly distributed in Central Asia, extending to northwestern China. Due to habitat fragmentation, overharvesting, and poor natural regeneration, this species has been designated as a Grade II national key protected species in China. **Methods**: To inform effective conservation strategies, we assessed the genetic diversity and population structure of *F. karelinii* using single nucleotide polymorphism (SNP) loci obtained via genotype-by-sequencing (GBS). A total of 87 individuals from 11 sampling sites across the Tacheng region in northern Xinjiang were genotyped, representing the species’ known extant distribution in China. **Results**: A relatively high level of genetic diversity (mean H_E_ = 0.1461) was detected in the species, and Bayesian assignment divided the sampled populations into two subtle but detectable genetic clusters. **Conclusions**: The relatively high diversity may be attributed to the edge-population effect and long-lived life-history traits, while the weak differentiation between the two groups may reflect restricted gene flow and genetic drift resulting from habitat isolation. Based on these findings, we propose conservation actions including immediate fencing of habitats to exclude livestock, ex situ germplasm collection separately from the two genetic groups as distinct management units, and cautious evaluation of potential germplasm introduction from Central Asia to broaden the genetic base while preserving local adaptation.

## 1. Introduction

Threatened plant species exhibit a range of traits that contribute to a higher risk of extinction than do other species [1,2]. Such species often have limited distribution, small population size, and highly fragmented habitats [3]. Due to the combined effects of environmental, demographic, and genetic stochasticity, small and isolated populations unavoidably suffer from genetic drift and inbreeding caused by small effective population sizes and limited gene flow [4,5,6]. Consequently, genetic diversity in small and isolated populations is eroded, which in turn reduces their viability and evolutionary potential in response to environmental change [7,8,9]. Thus, understanding the genetic diversity across the populations of threatened species is useful for developing conservation plans to preserve this diversity and reduce extinction risk [10,11].

Deserts, as a type of natural geographical landscape, are characterized by scarce precipitation and strong evaporative demand, with surface vegetation typically being sparse or even absent [12]. The Xinjiang region in northwestern China has an arid climate, characterized by widely distributed deserts and the Gobi, as well as interspersed mountains and basins [13]. Within this region, shrub vegetation is extensively distributed in deserts, the Gobi, piedmonts, and other landscapes [14]. The dominant plants in deserts of Xinjiang are extremely xerophytic semi-shrubs and shrubs, which are well adapted to the harsh desert conditions and form diverse desert plant communities [12]. Owing to the extremely harsh living environment and fragile habitats, desert ecosystems exhibit low stability, and the sparse vegetation has a weak capacity to resist natural disturbances [15]. The sparse vegetation is discontinuously distributed as patches, resulting in natural fragmentation and isolation among individuals of the same species [16].

*Fritillaria karelinii* Fisch. (Liliaceae) is a perennial herb. Its bulbs are used medicinally for moistening the lungs, resolving phlegm, and relieving cough and asthma, with efficacy equivalent to that of *F. pallidiflora* [17]. *Fritillaria karelinii* is mainly distributed in Central Asia, including Kazakhstan, Uzbekistan, Tajikistan, and Turkmenistan, and extends to Qapqal, Huocheng, Yining, and Tacheng counties in northwestern China, where it typically grows in *Artemisia*-dominated deserts or *Ferula* stands [18]. Due to its scarcity, *F. karelinii* has been listed in the List of Key Protected Wild Plants in Xinjiang and is designated as a Grade II national key protected species in China [19]. During two consecutive years of field survey, this species has been rarely observed in the Ili Valley, with only a few fragmented populations found in the Tacheng region. Suffering from both poor natural regeneration and overharvesting, *F. karelinii* has experienced population decline, placing it at increasing risk of extinction unless conservation efforts are strengthened.

Currently, information on the geographical distribution and genetic diversity of *Fritillaria karelinii* is virtually absent, and a population genetic study is urgently needed to preserve this valuable plant resource. With molecular markers at the whole-genome sequence level, a comprehensive assessment of species genetic diversity can be achieved [20]. Recently, reduced-representation genome sequencing has emerged as a powerful approach for obtaining SNPs, with advantages including independence from a reference genome, genome-wide coverage, high throughput, codominance, high stability, and substantially reduced costs [21]. This technique has been widely used in population genetics studies [22,23]. Here, we use GBS to obtain SNP markers, assess the genetic diversity and population structure of *F. karelinii*, and propose conservation strategies based on the findings.

## 2. Results

### 2.1. SNP Calling

Within the sequenced 87 individuals, the number of raw reads per individual ranged from 30,809,624 to 53,615,726, and the total number of bases per individual ranged from 4,436,585,856 bp to 7,720,664,544 bp. After excluding low-quality reads, the mean number of clean reads of all samples analyzed was 37,453,403, ranging from 26,359,192 to 50,446,846; the average depth of the samples was 12.25, ranging from 9.55 to 14.32. Of the initial 6,744,583 SNPs, 676,517 high-quality SNPs remained after filtering with dp3-miss0.2-maf0.01. To reduce the effect of linkage disequilibrium on species genetic structure, one SNP was randomly selected for each locus in the further analysis.

### 2.2. Genetic Diversity

As shown in *F. karelinii*, the mean expected heterozygosity (*He*) was 0.1461, ranging from 0.1447 to 0.1484; the mean observed heterozygosity (*Ho*) was 0.1353, ranging from 0.1330 to 0.1374; and the mean nucleotide diversity (*π*) was 2.5540 × 10^−5^, ranging from 2.4346 × 10^−5^ to 2.7645 × 10^−5^ (Table 1). Genetic differentiation between the populations (F_ST_) ranged from 0.0020 to 0.0178 (Table 2). AMOVA results showed that only 0.209% of the total variation occurs among groups, and 1.218% occurs among populations within groups, while the overwhelming majority (83.479%) resides within individuals (Table S1).

### 2.3. Genetic Structure

**Figure 1:**
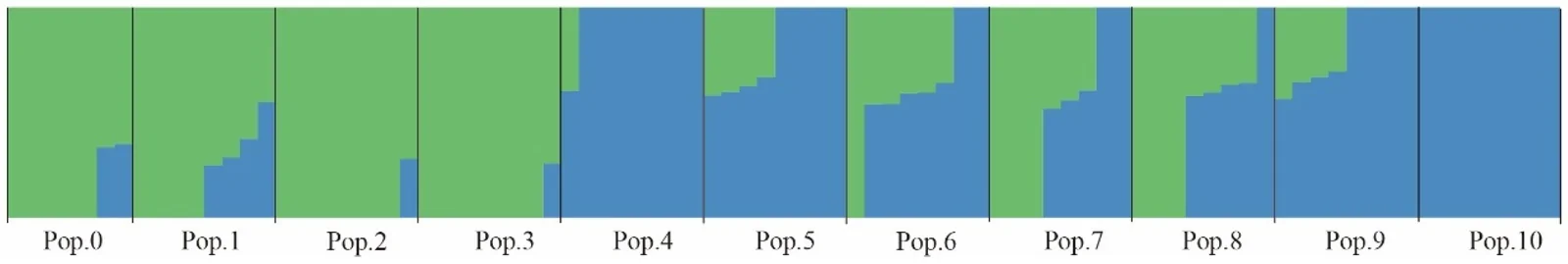
Genetic structure analysis of individuals from 11 *F. karelinii* populations using the program Admixture based on dataset D1. Two inferred groups are represented by two colors (green and blue). Each bar represents an individual with assignment probabilities to each group. The labels below the bar plot refer to the site code in Table 1. Populations 0–3 belong to Group 1, and populations 4–10 belong to Group 2.

**Figure 2:**
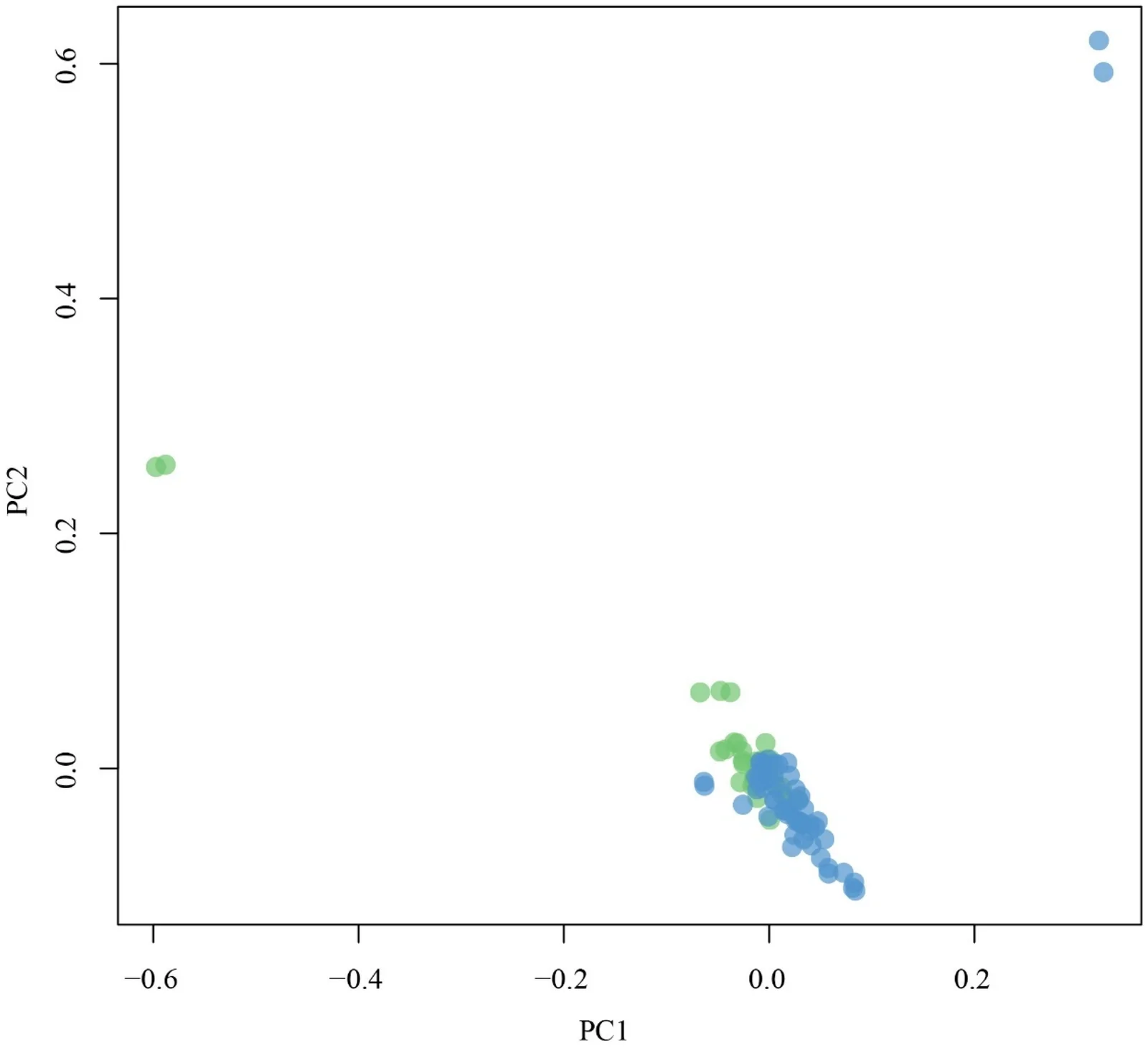
The PCA based on the SNP dataset D1. Green and blue colors correspond to Group 1 and 2 respectively.

For the original unfiltered dataset, the lowest CV error was observed at K = 2 (0.4332), leading to the selection of two genetic groups, with populations 0–3 assigned to Group 1 and populations 4–10 to Group 2 (Figure 1). Notably, a similarly highly clustered pattern was observed for both the LD-filtered and neutral datasets (Figure S1), where populations 0–3 and 4–10 consistently formed two genetic groups when K = 2 was applied. Principal component analysis (PCA) results were highly consistent between datasets D1, D2, and D3 (Figure S2). In each case, the majority of individuals clustered tightly together in a single group, with only a few individuals scattered along the principal components (Figure 2 and Figure S2). No significant correlation was detected between genetic distance and geographical distance across the entire distribution (r = 0.0625, *p* = 0.3611). The neighbor-joining (NJ) tree based on the SNP dataset showed a largely star-like topology with low bootstrap support for major clades, failing to separate individuals into distinct genetic clusters (Figure S3). This pattern is consistent with the PCA results, where most individuals clustered tightly together (Figure 2). In contrast, the Admixture analysis suggested a subtle genetic subdivision (K = 2). These apparent discrepancies likely arise from the extremely low genetic differentiation among populations (Table 2), which limits the resolution of distance-based and variance-based methods, while the model-based Admixture algorithm remains sensitive to incipient allele frequency differences. This combined evidence suggests a scenario of high overall genetic homogeneity with a weak emerging population structure.

We calculated genetic diversity indices for the two inferred groups. *He* was 0.1578 in group 1 and 0.1597 in group 2; *Ho* was 0.1349 in group 1 and 0.1356 in group 2; and *π* was 1.22 × 10^−5^ in group 1 and 1.23 × 10^−5^ in group 2.

## 3. Discussion

### 3.1. Genetic Diversity

Generally, species with a restricted geographic distribution have lower genetic diversity than those with a wide range [24,25]. Unfortunately, most population genetics studies of herbaceous plants were conducted using different types of molecular markers, making comparisons difficult. A comparable study on the alpine herb *Primula beesiana* using a similar reduced-representation sequencing method (ddRAD-seq) reported an average expected heterozygosity of 0.1083 [26]. The higher genetic diversity observed in *F. karelinii* (*He* = 0.1461) suggests a relatively high level of genetic diversity in *F. karelinii* populations.

Although the sampled populations in northwestern China are small and geographically restricted, *F. karelinii* has a wide distribution across Central Asia, particularly in Kazakhstan [17]. Our sampled populations, as peripheral populations at the edge of the species’ range, originated from these Central Asian sources [27]. The core distribution range of the species harbors larger and more continuous populations that may maintain high levels of standing genetic variation. Historical gene flow from these core populations to the edge could have replenished genetic diversity in the peripheral populations, enabling them to retain a substantial proportion of the species’ overall genetic diversity despite their limited local population sizes. This ‘edge-population’ effect offers a plausible explanation for the relatively high genetic diversity observed in our study, although this hypothesis remains to be rigorously tested with broader sampling encompassing the Central Asian core distribution [28,29].

Additionally, as a perennial herb with a bulbous life form, *F. karelinii* exhibits overlapping generations and a potentially extended life span. These life-history traits can buffer the loss of genetic diversity by reducing the rate of genetic drift compared to annual species [30]. Similarly, longevity and overlapping generations may render perennial species less sensitive to the detrimental effects of genetic bottlenecks caused by habitat fragmentation [31].

### 3.2. Genetic Structure

Understanding the genetic structure of threatened species is essential for defining conservation units and developing effective conservation strategies [32]. Genetic structure is influenced by multiple factors, including genetic drift within isolated populations [33], limited gene flow between populations [34,35], historical founder effects [36,37], and life-history traits such as pollination mode and seed dispersal mechanisms [38,39]. Based on Admixture, the 11 sampling sites of *F. karelinii* were divided into two subtle but detectable genetic clusters, a northern group (populations 0–3) and a southern group (populations 4–10). Even though the species occupies a very limited distribution range, the habitats occupied by each genetic group differ in type. The northern group inhabits desert steppe, with *Agropyron cristatum* and *Artemisia* species as the constructive species. In contrast, the southern group occurs in desert shrubland steppe, with *Oreosalsola laricifolia* as the constructive species. Between the two groups lies *Achnatherum splendens* steppe. Although *F. karelinii* produces showy, insect-pollinated flowers, actual pollen-mediated gene flow appears limited to short distances due to habitat isolation. On the other hand, seed dispersal of *F. karelinii* is likely limited by gravity, with seeds usually remaining clustered near the parent plant. Consequently, although some gene flow may occur between the two groups, it is largely constrained, allowing genetic differentiation to be maintained.

Moreover, populations of *F. karelinii* are scattered across different hills in the desert, generally isolated from each other and small in size (fewer than 30 individuals per population). Small and isolated populations experience increased random genetic drift, which leads to the random fixation of alleles [5,40]. Consequently, allelic composition among populations diverges, promoting population differentiation [41,42]. Additionally, the occurrence of *F. karelinii* in the Tacheng region represents a range expansion from its core distribution in Central Asia. During the initial colonization process, it is unlikely that a large number of individuals migrated simultaneously. Instead, small numbers of seeds likely arrived at different times and established populations on different hills. Because the species reproduces primarily by seed, each colonization event might carry only a subset of the genetic diversity from the source population. Such founder effects would have immediately created initial genetic differences among the newly colonized populations, which could persist if subsequent gene flow is limited.

It is noteworthy that the NJ tree and PCA did not fully recapitulate the two-group subdivision inferred by Admixture. This discordance is not unexpected in population genomics, particularly when overall genetic divergence is exceedingly low. NJ trees rely on cumulative pairwise distances; when inter-population distances do not substantially exceed intra-population distances, monophyletic groupings cannot be reliably resolved. Likewise, PCA captures the largest axes of variance, which in this case are dominated by individual-level variation rather than inter-group differentiation. Conversely, Admixture employs a likelihood-based ancestry model that can detect minor but cumulative allele frequency differences across numerous loci, thereby revealing a cryptic or incipient subdivision. In our study, this subtle subdivision aligns with habitat heterogeneity; northern populations (0–3) occur in desert steppe, while southern populations (4–10) inhabit desert shrubland steppe, suggesting that ecological factors may be exerting weak divergent selection or reinforcing limited gene flow, even though the overall genomic landscape remains largely homogeneous.

### 3.3. Conservation Implications

Despite the relatively high genetic diversity observed, the species’ limited distribution and small population sizes suggest a pessimistic long-term outlook. Urgent conservation actions are required to secure its long-term persistence. Although the Flora of China records *F. karelinii* in Yining, Qapqal, and Huocheng counties of the Ili Valley, our recent field surveys have failed to locate any individuals there. Therefore, the populations in the Tacheng region currently may represent the only known extant populations of this species in China. These remaining populations are situated within local pastoral lands, where the species grows as a spring ephemeral. Each spring, the species faces severe grazing pressure and is often consumed before it reaches flowering and fruiting stages, severely impairing its natural regeneration. Thus, the highest priority, a recommendation derived directly from our field observations of grazing pressure and the consequent failure of plants to complete their life cycle, is to protect the habitats of all Tacheng populations. Setting up a fence to exclude livestock should be implemented immediately to allow the plants to complete their life cycle. Secondly, ex situ conservation should be established through a germplasm collection. Although the overall genetic differentiation is low (as reflected by F_ST_, Amova, and PCA results), our genetic analyses did reveal two subtle but detectable genetic clusters (northern and southern groups) within the Tacheng region, corresponding to different habitat types. Given this genetic signal and the ecological distinction between the two areas, we suggest that each be considered a separate management unit. Seeds should be collected separately from the northern and southern groups to capture the full range of genetic diversity when establishing a germplasm nursery. The seedlings produced can be reintroduced into the original habitats or transplanted to reconstruct populations in suitable sites within the Ili Valley. Thirdly, international collaboration should be strengthened to introduce germplasm from the core distribution area in Central Asia, which would broaden the genetic base and support large-scale multiplication efforts. This latter suggestion is a general conservation recommendation that, if pursued, should be carefully evaluated to prevent potential disruption of local genetic structure and local adaptation.

## 4. Materials and Methods

### 4.1. Sampling

**Figure 3:**
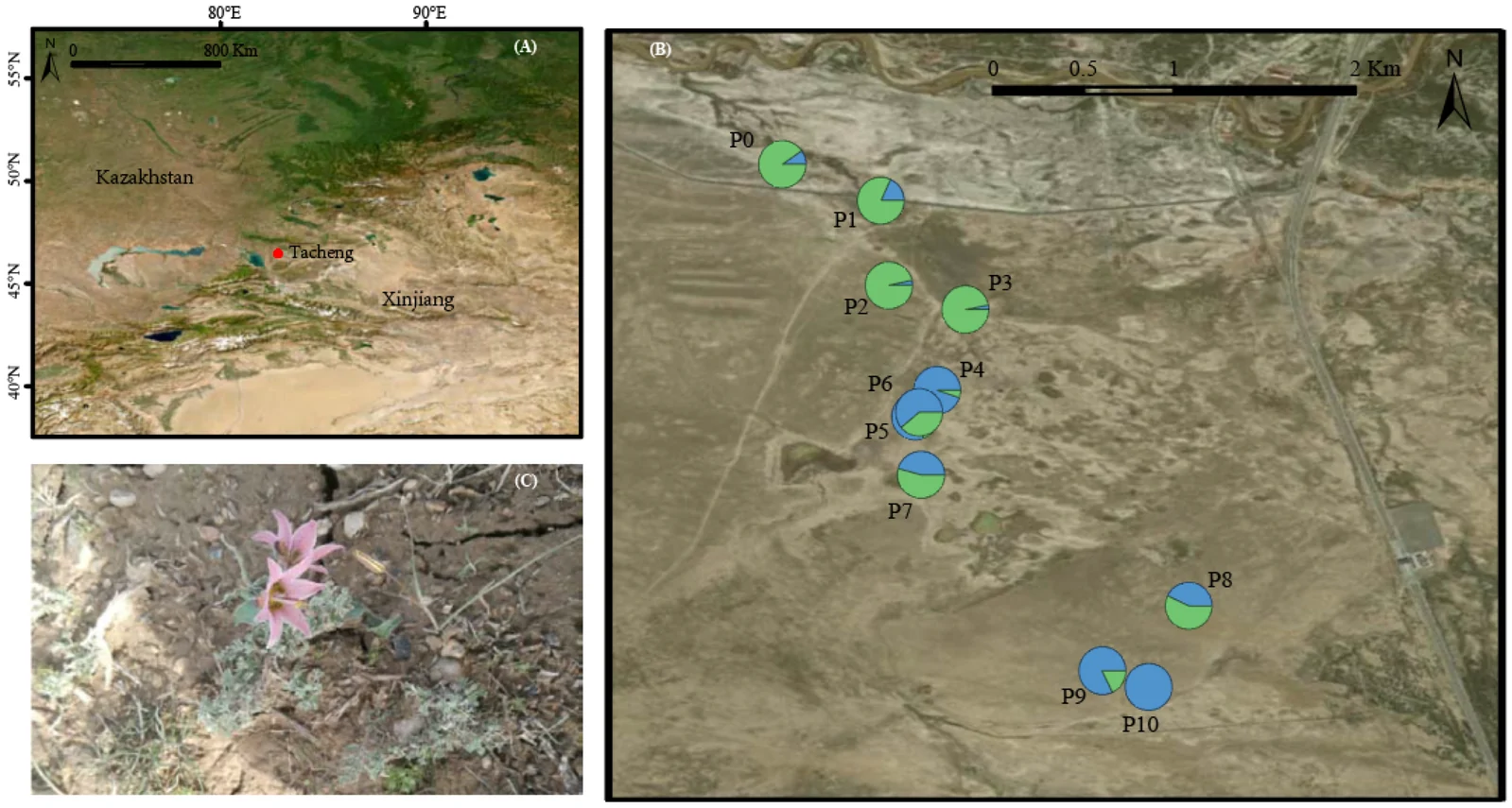
Geographic location of *F. karelinii* populations in the northern Balruk Mountains in northwestern China: (**A**) sampling locations on the geographic background map; (**B**) genetic ancestry proportions for each population inferred from Admixture analysis (colors represent distinct genetic clusters, corresponding to Figure 1, with green and blue colors corresponding to Group 1 and 2 respectively); (**C**) photograph of a representative *F. karelinii* individual. Population IDs correspond to those listed in Table 1.

A total of 87 individuals from 11 sampling sites of *Fritillaria karelinii* were sampled, covering almost the entire geographic distribution of the species in the Tacheng region in northwestern China. These 11 sampling sites are geographically restricted within the Tacheng region and are referred to as ‘populations’ throughout the text solely for convenience in population genetic analyses, though they represent sampling localities rather than isolated biological populations with clear reproductive boundaries. Eight individuals were sampled from each of ten populations and seven from one population (Figure 3, Table 1). To avoid sampling clonally propagated ramets, we sampled only one individual per cluster, with a minimum distance of 30 m between clusters. Fresh leaves were collected from each individual and dried in silica gel. The leaves were stored at 4 °C.

### 4.2. GBS Library Preparation

Using the DNeasy Plant Mini Kit (Qiagen, Hilden, Germany), total genomic DNA was extracted from 87 samples following the manufacturer’s instructions. Two high-fidelity restriction enzymes, namely NlaIII and EcorI, were selected to digest DNA [43]. For each individual, the digested DNA was uniquely barcoded, and the barcoded samples were then pooled. The pooled libraries were amplified by multiplexed polymerase chain reactions (PCRs), and the products were purified using VAHTSTM DNA Clean Beads (Vazyme Biotech Co., Nanjing, China). The pooled library, containing 87 barcoded individuals, was sequenced on Illumina HiSeq PE150 with 2 × 150 paired-end reads. The construction of the GBS library and sequencing were performed by Novogene Bioinformatics Technology Co., Ltd., Beijing, China.

### 4.3. SNP Calling

Raw reads were demultiplexed and processed following the standard GBS analysis pipeline, with adapter trimming performed using an in-house Perl script [44]. Reads containing adapter sequences, along with paired-end reads where either read had ≥50% low-quality bases (quality value ≤ 5) or ≥10% unidentified nucleotides, were filtered out. In addition, putative duplicate reads and reads that lacked an intact restriction site were discarded. The proportion of high-quality reads with a Q30 score (99.9% accuracy) and the guanine–cytosine (GC) content were calculated for quality control. The GBS reads were processed with STACKS v2.30 [45]. Tags were generated for each sample, and the sample with the highest tag count was selected as the reference to construct a catalog of consensus stacks. High-quality sequencing reads were aligned to the reference genome using BWA with parameters “mem-t 4-K 32-M”, and the resulting alignments were sorted using SAMTOOLS v1.24 [46]. SNP loci were detected using SAMTOOLS and then filtered according to the following criteria: SNPs with a quality score below Q20 (sequencing error rate > 1%) were discarded; only SNPs with a coverage depth > 3 were retained; SNPs with a missing genotype rate > 20% were removed; and SNPs with a minor allele frequency (MAF) < 0.01 were excluded. The remaining high-quality SNPs (dataset D1) were used for subsequent analyses. To mitigate the impact of linkage disequilibrium, we retained a single SNP per contig of the pseudo-reference genome (dataset D2) and further removed outlier SNPs potentially under selection as determined by BayeScan v2.1 to retain only neutral loci (dataset D3).

### 4.4. Genetic Diversity and Structure

Admixture analysis, NJ tree, and PCA were performed to estimate the genetic structure of *Fritillaria karelinii* populations. Admixture analysis was performed using Admixture v 1.30 [47], assuming unlinked loci. To determine the optimal number of genetically distinct groups (K), 10 replicates were run, and the mean cross-validation error was calculated for each K (from 2 to 10). The optimal K was chosen as the one with the lowest mean cross-validation error. The NJ tree was constructed based on the pairwise individual genetic distance (Dij) matrix using TreeBest v 1.9.2 [48], with 1000 bootstrap replicates.

PCA was performed using GCTA v1.24.2 [49]. The first two principal components were then compared with geographic distances using Procrustes analysis implemented in the R package ‘vegan’ v1.8-5 [50], which revealed a relationship between genetic structure and geographic distribution. To compare differences in genetic structure across datasets, we performed admixture analysis and PCA on all datasets.

The following standard parameters of genetic diversity at the population and group levels were estimated: *He*, *H_O_*, *π*, and pairwise F_ST_ between populations, using the populations module in Stacks v1.46 [45]. To examine the effect of isolation by distance within *F. karelinii*, a Mantel test was performed in ARLEQUIN v3.01, and to estimate genetic differentiation within and among populations, AMOVA was also performed in this program [51]. The genetic distance between populations was represented by pairwise F_ST_, and the geographic distances between populations were calculated in GEODIS v2.5 [52].

## Figures and Tables

**Table 1:** Sampling information of *F. karelinii* populations and genetic diversity based on SNP data.

Population ID	Altitude	Sample Size	*He*	*Ho*	*π* (10^−5^)
Pop.0	412	7	0.1455	0.1360	2.6524
Pop.1	347	8	0.1447	0.133	2.4402
Pop.2	352	8	0.1457	0.1348	2.4541
Pop.3	344	8	0.1472	0.1354	2.6258
Pop.4	349	8	0.1484	0.1374	2.7645
Pop.5	340	8	0.1467	0.1366	2.5275
Pop.6	342	8	0.1451	0.1362	2.4346
Pop.7	337	8	0.1471	0.1357	2.6009
Pop.8	365	8	0.1447	0.1334	2.4643
Pop.9	365	8	0.1460	0.1353	2.5458
Pop.10	353	8	0.1464	0.1340	2.5843
Group1		31	0.1578	0.1349	1.2226
Group2		56	0.1597	0.1356	1.2324

Group 1 includes populations 0–3, and Group 2 includes populations 4–10.

**Table 2:** Pairwise estimated values of F_ST_ (below diagonal) between the populations of *F. karelinii*.

	Pop.0	Pop.1	Pop.2	Pop.3	Pop.4	Pop.5	Pop.6	Pop.7	Pop.8	Pop.9	Pop.10
Pop.0											
Pop.1	0.0043										
Pop.2	0.0042	0.0056									
Pop.3	0.0106	0.0112	0.0058								
Pop.4	0.0146	0.0156	0.0121	0.0168							
Pop.5	0.0110	0.0111	0.0088	0.0147	0.0140						
Pop.6	0.0072	0.0071	0.0050	0.0099	0.0121	0.0067					
Pop.7	0.0079	0.0084	0.0078	0.0128	0.0145	0.0103	0.0067				
Pop.8	0.0076	0.0080	0.0078	0.0123	0.0149	0.0118	0.0024	0.0074			
Pop.9	0.0061	0.0073	0.0058	0.0118	0.0131	0.0100	0.0058	0.0051	0.0021		
Pop.10	0.0100	0.0102	0.0105	0.0160	0.0178	0.0136	0.0087	0.0098	0.0072	0.0020	

## Data Availability

Illumina sequence read data obtained in this study are available through NCBI BioProject PRJNA1490068.

## Supplementary Materials

The following supporting information can be downloaded at https://www.mdpi.com/article/10.3390/genes17091010/s1, Figure S1: Genetic structure analysis of 11 *F. karelinii* populations using Admixture, based on dataset D2 (A) and D3 (B); Figure S2: The PCA based on the SNP dataset D2 (A) and D3 (B); Figure S3: The NJ tree for 87 *F. karelinii* individuals; Table S1: Results of the analysis of molecular variance for 11 populations of *F. karelinii* based on GBS-seq SNP data.
