# Supplementary material for: Genetic Diversity and Structure of an Endangered Medicinal Plant Species in the Northern Balruk Mountains: Implications for Conservation
Source: Genes (Basel). 2026 Aug 26;17(9):1010. doi: 10.3390/genes17091010 (PMC13606599; doi:10.3390/genes17091010)
Supplement: Supplementary file 1 [file genes-17-01010-s001.zip › Table S1.pdf]

Table S1. Results of the analysis of molecular variance for 11 populations of *F. karelinii* based on GBS-seq SNP data.

**Source of Variation**	**Sum of Squares**	**Variance Components**	**Percentage Variation**
Among groups	79583.292	114.622	0.209
Among populations within groups	643884.881	668.212	1.218
Among individuals within populations	4058832.209	8279.186	15.094
Within individuals	3531875.500	45788.950	83.479
Total	8314175.883	54850.971	
