# Supplementary material for: Genetic Diversity and Structure of an Endangered Medicinal Plant Species in the Northern Balruk Mountains: Implications for Conservation
Source: Genes (Basel). 2026 Aug 26;17(9):1010. doi: 10.3390/genes17091010 (PMC13606599; doi:10.3390/genes17091010)
Supplement: Supplementary file 1 [file genes-17-01010-s001.zip › Fig. S1.pdf]

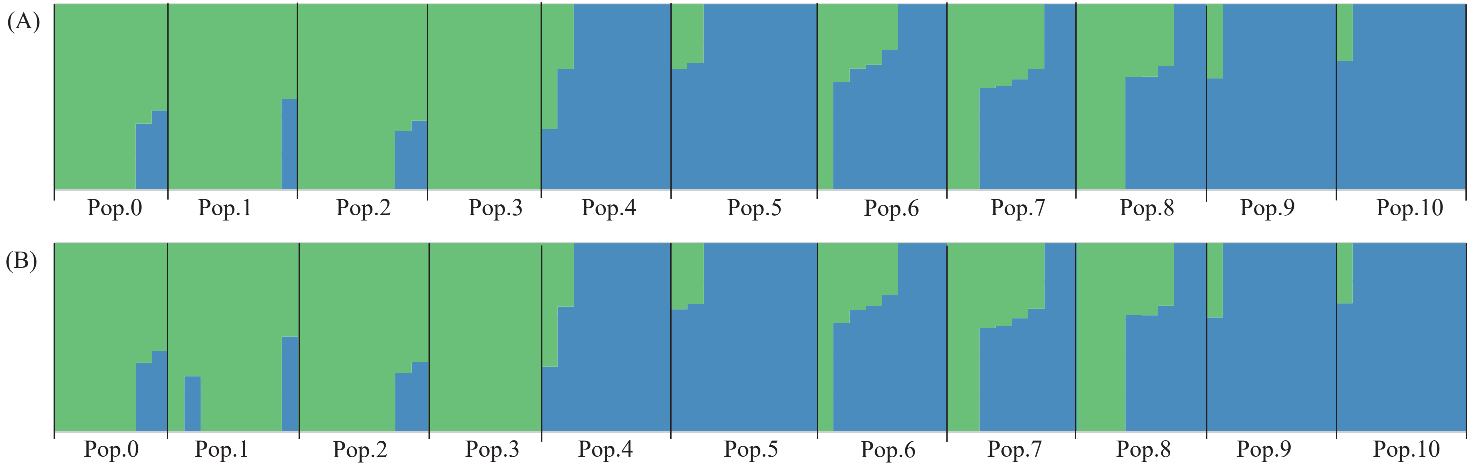

(A)
Pop.0
Pop.1
Pop.2
Pop.3
Pop.4
Pop.5
Pop.6
Pop.7
Pop.8
Pop.9
Pop.10
(B)
Pop.0
Pop.1
Pop.2
Pop.3
Pop.4
Pop.5
Pop.6
Pop.7
Pop.8
Pop.9
Pop.10
