# Supplementary material for: Genetic Diversity and Structure of an Endangered Medicinal Plant Species in the Northern Balruk Mountains: Implications for Conservation
Source: Genes (Basel). 2026 Aug 26;17(9):1010. doi: 10.3390/genes17091010 (PMC13606599; doi:10.3390/genes17091010)
Supplement: Supplementary file 1 [file genes-17-01010-s001.zip › Fig. S2.pdf]

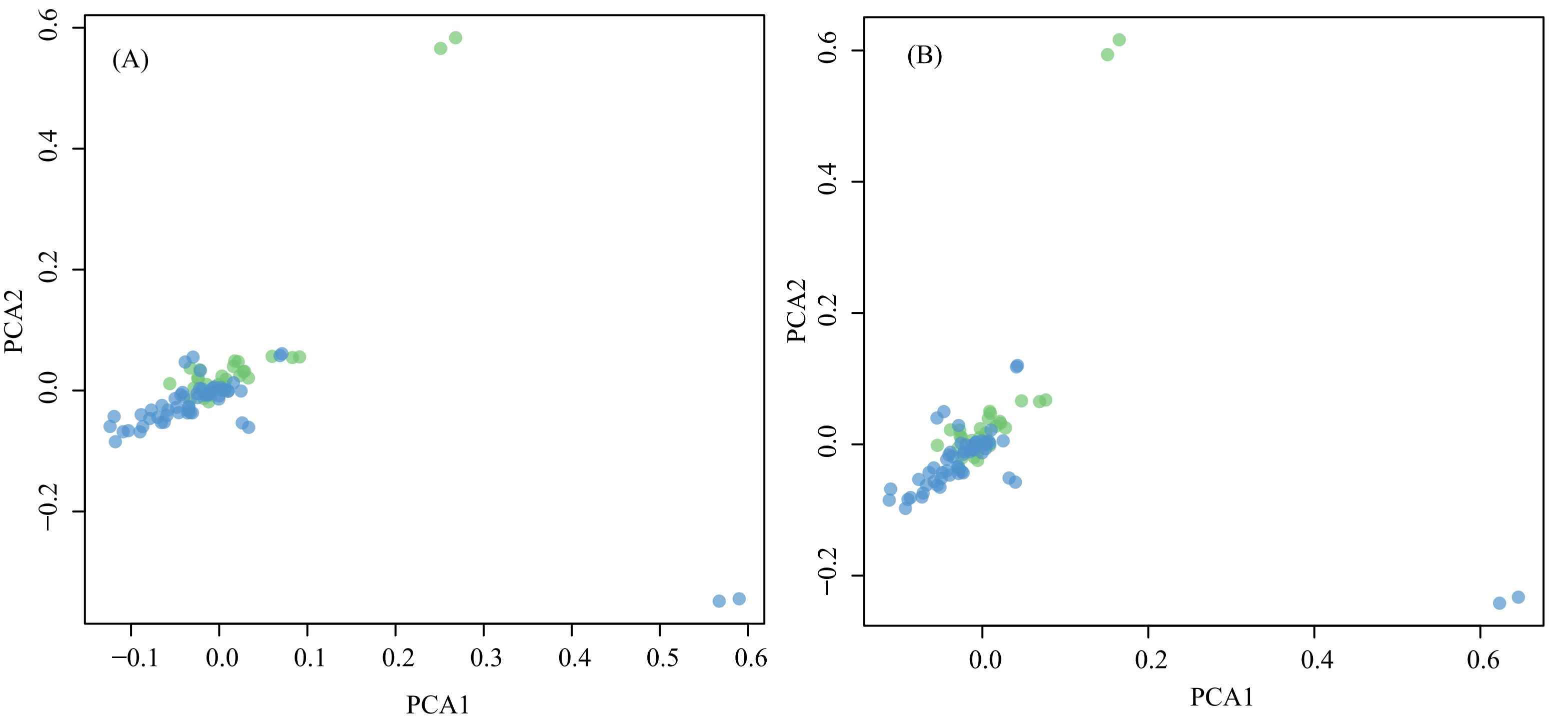
(A)
PCA1
PCA2
−0.1
0.0
0.1
0.2
0.3
0.4
0.5
0.6
−0.2
0.0
0.2
0.4
0.6
(B)
PCA1
PCA2
0.0
0.2
0.4
0.6
−0.2
0.0
0.2
0.4
0.6
