# Supplementary material for: Genetic Diversity and Structure of an Endangered Medicinal Plant Species in the Northern Balruk Mountains: Implications for Conservation
Source: Genes (Basel). 2026 Aug 26;17(9):1010. doi: 10.3390/genes17091010 (PMC13606599; doi:10.3390/genes17091010)
Supplement: Supplementary file 1 [file genes-17-01010-s001.zip › Fig. S3.pdf]

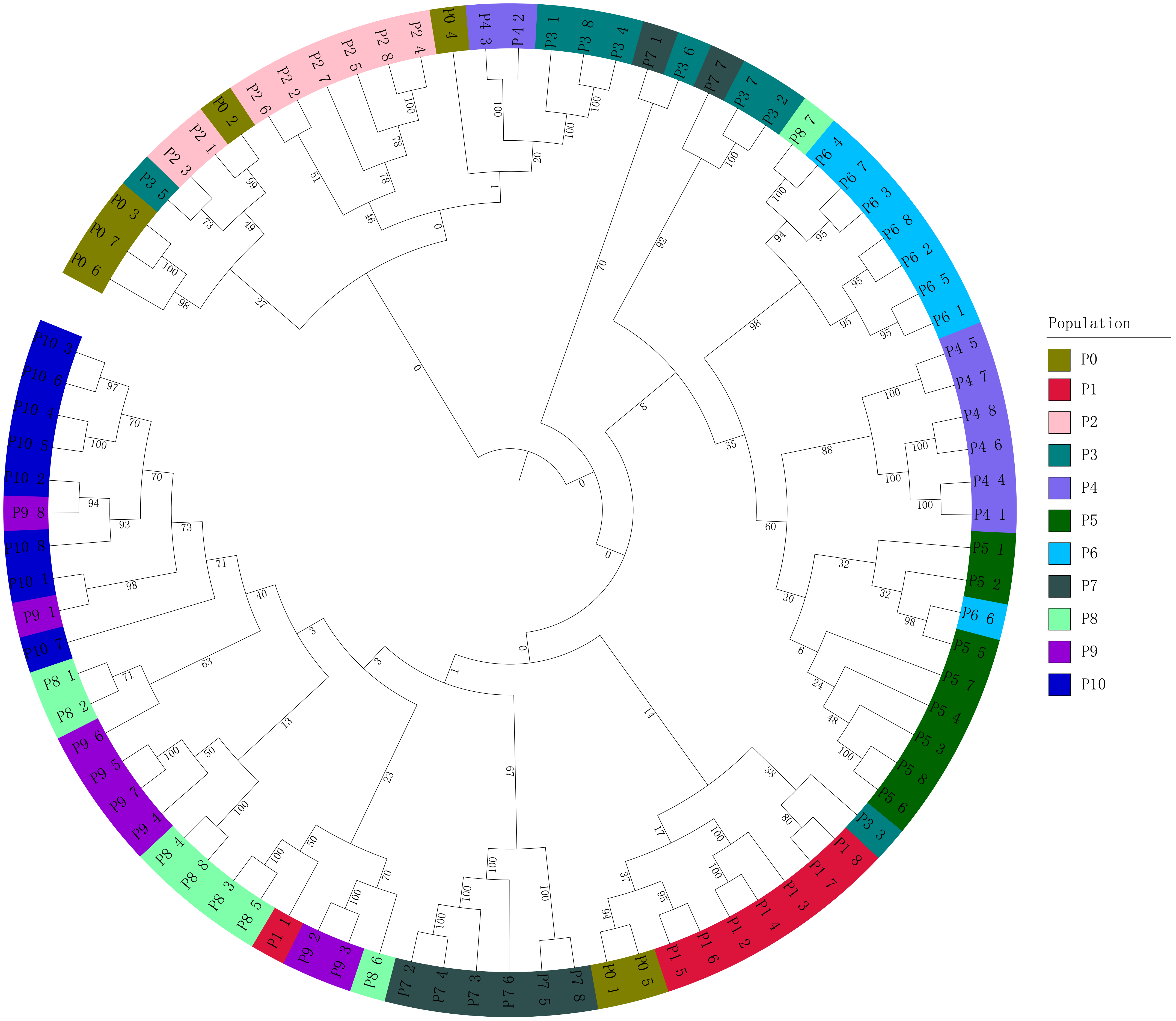

Population
P0
P1
P2
P3
P4
P5
P6
P7
P8
P9
P10
